# Synthesis, crystal structure and Hirshfeld surface analysis of *tert*-butyl 4-[4-(di­fluoro­meth­oxy)phen­yl]-2,7,7-trimethyl-5-oxo-1,4,5,6,7,8-hexa­hydro­quinoline-3-carboxyl­ate

**DOI:** 10.1107/S2056989023005455

**Published:** 2023-06-30

**Authors:** Ezgi Pehlivanlar, Sema Öztürk Yıldırım, Rahime Şimşek, Mehmet Akkurt, Ray J. Butcher, Ajaya Bhattarai

**Affiliations:** a Department of Pharmaceutical Chemistry, Faculty of Pharmacy, Hacettepe University, 06100 Ankara, Türkiye; b Department of Pharmaceutical Chemistry, Faculty of Pharmacy, Karadeniz Technical University, 61000 Trabzon, Türkiye; c Department of Physics, Faculty of Science, Eskisehir Technical University, Yunus Emre Campus 26470 Eskisehir, Türkiye; d Department of Physics, Faculty of Science, Erciyes University, 38039 Kayseri, Türkiye; e Department of Chemistry, Howard University, Washington DC 20059, USA; f Department of Chemistry, M.M.A.M.C (Tribhuvan University), Biratnagar, Nepal; Venezuelan Institute of Scientific Research, Venezuela

**Keywords:** crystal structure, hydrogen bonds, van der Waals forces, C—H⋯F inter­actions, Hirshfeld surface analysis

## Abstract

The 1,4-di­hydro­pyridine ring of the title compound, C_24_H_29_F_2_NO_4_, adopts a distorted boat conformation, while the cyclo­hexene ring is in an almost twist-boat conformation. In the crystal, N—H⋯O and C—H⋯O hydrogen bonds as well as C—H⋯π inter­actions connect mol­ecules, forming layers parallel to the (100) plane. These layers are linked by van der Waals forces and C—H⋯F inter­actions, which consolidate the crystal structure. Hirshfeld surface analysis shows the major contributions to the crystal packing are from H⋯H (54.1%), F⋯H/H⋯F (16.9%), O⋯H/H⋯O (15.4%) and C⋯H/H⋯C (12.6%) contacts.

## Abstract 2

In the crystal, mol­ecules are connected *via* N—H⋯O and C—H⋯O hydrogen bonds and C—H⋯π inter­actions, forming layers parallel to the (100) plane. van der Waals forces and C—H⋯F inter­actions connect these layers, consolidating the crystal structure.

## 1. Chemical context

Inflammation is the natural and basic response of an organism to signals from tissue damage or pathogenic infections. In this way, the integrity of the organism is preserved. Chronic diseases that cause death and economic losses in the world are constantly increasing. It has been found that chronic diseases occur through inflammation-mediated mechanisms. In recent years, it has been proven that cardiovascular diseases, cancer, diabetes mellitus, chronic kidney disease, non-alcoholic fatty liver disease, autoimmune and neurodegenerative diseases are caused by inflammation. In this context, managing inflammatory mediators and inflammatory processes can be a treatment method for many chronic diseases (Furman *et al.*, 2019 ▸; Tu *et al.*, 2022 ▸).

**Figure 1:**
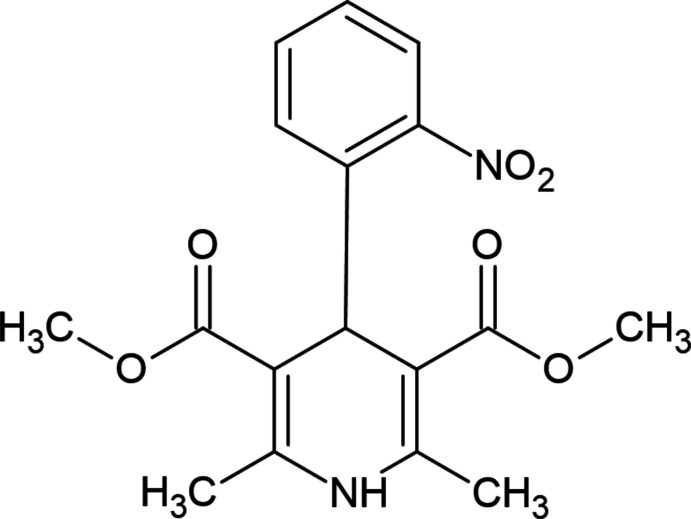
Structure of nifedipine.

Chronic or local inflammation first occurs with the activation of immune system cells such as cytokines, proteases, chemokines, oxygen-independent radicals, which generate signals from damaged cells or pathogens that are dangerous to the tissue. The immune system cells released in the circulatory system increase the pro-inflammatory response and reach the infected tissue area, but if this response is insufficient or excessive, the balance of the immune system is disturbed. This imbalance causes an excessive amount of distress signals and local or systemic tissue damage. This defect in the immune response causes the inflammation to change from acute to chronic, and the disease progresses and results in death. A better understanding of inflammation and its processes enables the discovery of new and effective therapeutic ways to target and regulate inflammation. Drug therapy is widely used for the treatment of inflammation. Therefore, there is a need for new mol­ecules that are more active and have minimal side effects (Tu *et al.*, 2022 ▸). The 1,4-DHP ring, which is a partially saturated derivative of the pyridine ring, is involved in the structure of many bioactive compounds. Nifedipine, which has a 1,4-DHP structure, was introduced as an anti­hypertensive treatment about 50 years ago (Fig. 1 ▸). The therapeutic success of nifedipine has led to the preparation of analogue derivatives. In this ongoing process, various compounds such as amlodipine and benidipine, which have a 1,4-DHP structure, are used as antihypertensives. Studies have shown that the 1,4-DHP ring has various activities such as neuroprotective, anti­platelet, anti-ischemic, anti-Alzheimer’s, anti­tuberculer, anti­ulcer and anti­cancer (Khot *et al.*, 2021 ▸; Abdelwahab *et al.*, 2022 ▸).

The hexa­hydro­quinoline ring system is obtained by condensing 1,4-DHP with cyclo­hexane. This ring system also has a variety of pharmacological activities such as calcium channel antagonist, anti­cancer, anti­microbial, anti-Alzheim­er’s. In current studies, 1,4-DHP derivatives and condensed analogues were found to be effective inflammation mediators of chronic inflammation in addition to their various biological activities. 
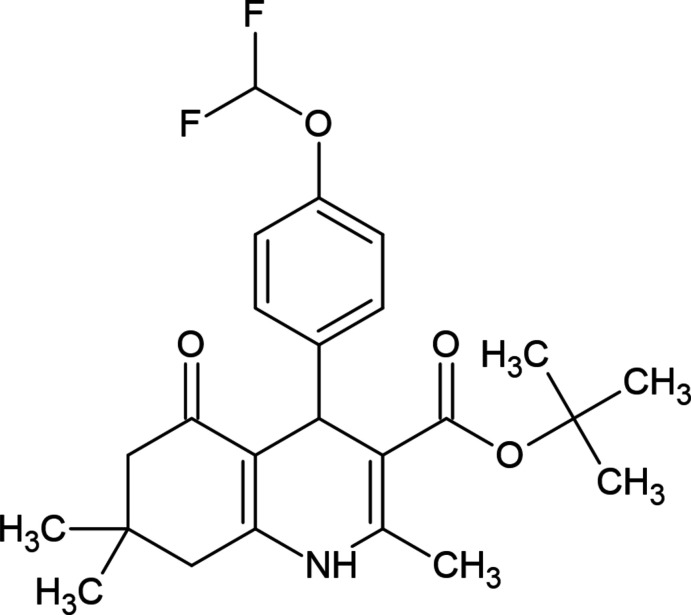


In this study, the title compound, *tert*-butyl 4-[4-(di­fluoro­meth­oxy)phen­yl]-2,7,7-trimethyl-5-oxo-1,4,5,6,7,8-hexa­hydro­quinoline-3-carboxyl­ate was obtained by using modified Hantzsch one-pot synthesis (Ghosh *et al.*, 2013 ▸). The reaction of 4-di­fluoro­meth­oxy­benzaldehyde with 5,5-di­methyl­cyclo­hexane-1,3-dione and *tert*-butyl aceto­acetate gives the target compound in methanol in the presence of ammonium acetate as nitro­gen source (Çetin *et al.*, 2022 ▸). The structure of the compound was elucidated by IR, ^1^H-NMR, ^13^C-NMR and HRMS analysis. X-ray analysis was undertaken to determine the crystal structure. Biological activity tests will be conducted in independent studies to determine the inhibition potential of inflammation mediators.

## 2. Structural commentary

**Figure 2:**
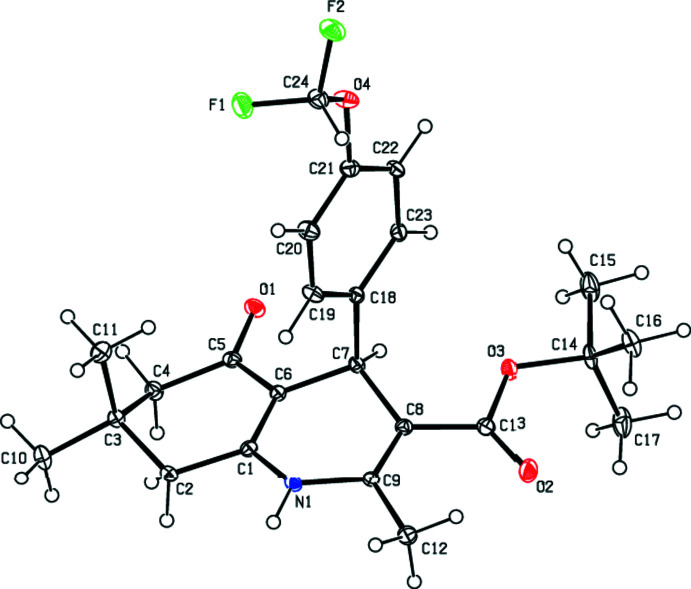
View of the title mol­ecule. Displacement ellipsoids are drawn at the 30% probability level.

As seen in Fig. 2 ▸, the 1,4-di­hydro­pyridine ring (N1/C1/C6–C9) of the title compound adopts a distorted boat conformation [puckering parameters (Cremer & Pople, 1975 ▸) are *Q*_T_ = 0.2940 (18) Å, θ = 72.1 (4)° and φ = 182.9 (4)°], while the cyclo­hexene ring (C1–C6) has an almost twist-boat conformation [puckering parameters are *Q*_T_ = 0.4617 (19) Å, θ = 124.5 (2)° and φ = 313.8 (3)°]. The 4-[4-(di­fluoro­meth­oxy]phenyl ring (C18–C23) makes a dihedral angle of 89.88 (7)° with the mean plane of the quinoline ring system [N1/C1–C9; maximum deviation = 0.358 (2) Å for C4]. The geometrical parameters of the title compound are in agreement with those reported for similar compounds in the *Database survey* section.

## 3. Supra­molecular features and Hirshfeld surface analysis

**Figure 3:**
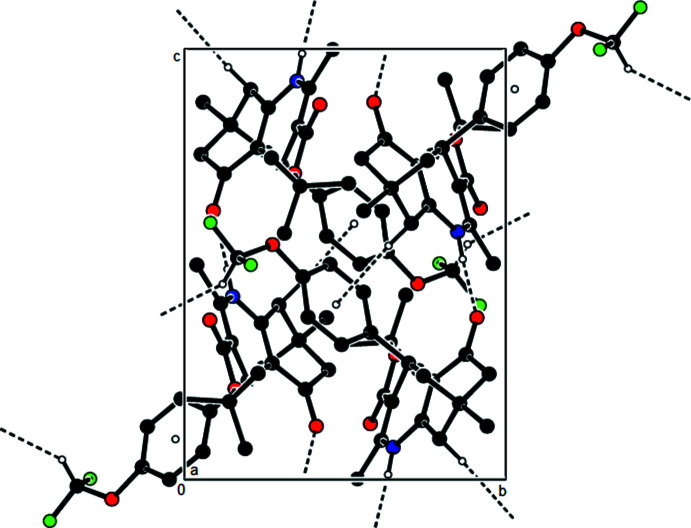
A view of the mol­ecular packing of the title compound along the *a* axis by the N—H⋯O, C—H⋯O hydrogen bonds and C—H⋯π inter­actions (dashed lines).

**Figure 5:**
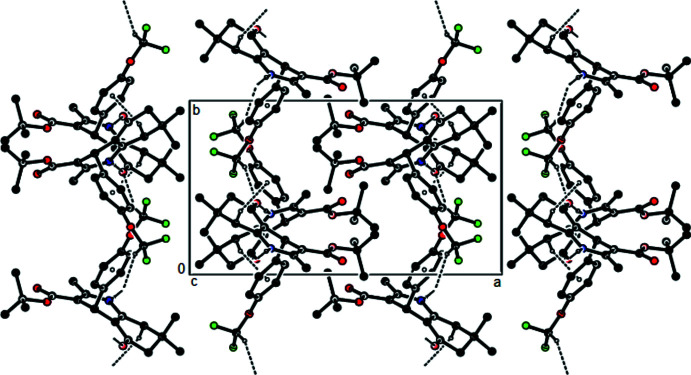
View of the mol­ecular packing along [001]. Hydrogen bonds are shown as dashed lines.

The mol­ecules in the crystal are connected by N—H⋯O and C—H⋯O hydrogen bonds, as well as C—H⋯π inter­actions, resulting in the formation of layers parallel to the (100) plane (see Table 1 ▸; Figs. 3 ▸ and 4 ▸). These layers are linked by van der Waals forces and C—H⋯F inter­actions, which consolidate the crystal structure (Fig. 5 ▸).

**Figure 6:**
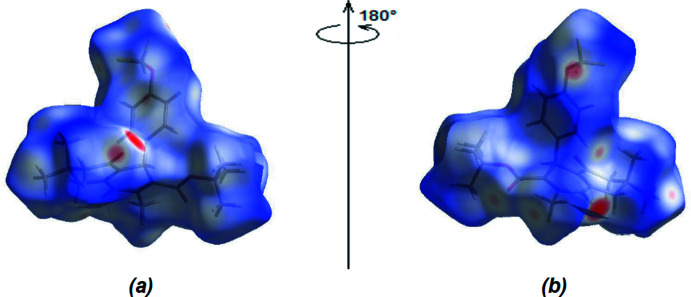
(*a*) Front and (*b*) back views of the three-dimensional Hirshfeld surface for the title compound.

The Hirshfeld surfaces and their corresponding two-dimensional fingerprint plots were calculated using the *Crystal Explorer 17.5* (Spackman *et al.*, 2021 ▸) software package. The *d*_norm_ surfaces are mapped over a fixed colour scale from −0.5814 (red) to +1.6362 (blue) a.u. Red spots on the surface correspond to N⋯H/H⋯N and O⋯H/H⋯O inter­actions (Tables 1 ▸ and 2 ▸; Fig. 6 ▸ *a*,*b*).

**Figure 7:**
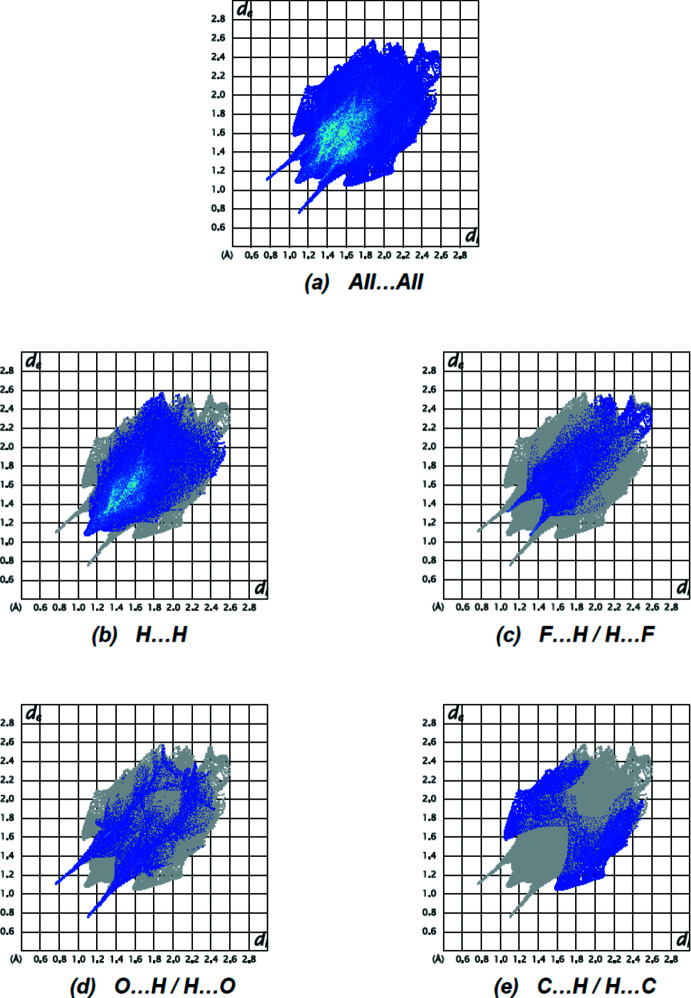
The two-dimensional fingerprint plots for the title compound showing (*a*) all inter­actions, and delineated into (*b*) H⋯H, (*c*) F⋯H/H⋯F, (*d*) O⋯H/H⋯O and (*e*) C⋯H/H⋯C inter­actions. The *d*_i_ and *d*_e_ values are the closest inter­nal and external distances (in Å) from given points on the Hirshfeld surface.

Fingerprint plots of the most important non-covalent inter­actions for the title compound are shown in Fig. 7 ▸. The major contributions to the crystal packing are from H⋯H (54.1%), F⋯H/H⋯F (16.9%), O⋯H/H⋯O (15.4%) and C⋯H/H⋯C (12.6) contacts. N⋯H/H⋯N (0.5%), F⋯N/N⋯F (0.3%) and F⋯F (0.2%) contacts, which contribute less than 1%, are not shown in Fig. 7 ▸.

## 4. Database survey

A search of the Cambridge Structural Database (CSD, Version 5.42, update of September 2021; Groom *et al.*, 2016 ▸) for similar structures with the 1,4,5,6,7,8-hexa­hydro­quinoline group showed that the nine most closely related to the title compound are WEZJUK (Yıldırım *et al.*, 2023 ▸), ECUCUE (Yıldırım *et al.*, 2022 ▸), LOQCAX (Steiger *et al.*, 2014 ▸), NEQMON (Öztürk Yıldırım *et al.*, 2013 ▸), PECPUK (Gündüz *et al.*, 2012 ▸), IMEJOA (Linden *et al.*, 2011 ▸), PUGCIE (Mookiah *et al.*, 2009 ▸), UCOLOO (Linden *et al.*, 2006 ▸) and DAYJET (Linden *et al.*, 2005 ▸). In all these compounds, mol­ecules are linked by N—H⋯O hydrogen bonds. Furthermore, C—H⋯O hydrogen bonds in WEZJUK, ECUCUE, NEQMON, IMEJOA and PUGCIE and C—H⋯π inter­actions in WEZJUK and ECUCUE were also observed.

## 5. Synthesis and crystallization

**Figure 8:**
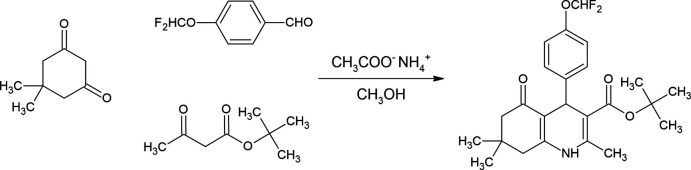
Synthetic scheme.

The target compound was synthesized by refluxing 5,5-di­methyl­cyclo­hexane-1,3-dione (1 mmol), 4-di­fluoro­meth­oxy­benzaldehyde (1 mmol), *tert*-butyl­aceto­acetate (1 mmol) and ammonium acetate (5 mmol) for 8 h in absolute methanol (10 ml). The reaction mixture was monitored by TLC, and after completion of the reaction was cooled to room temperature. The obtained precipitate was filtered and recrystallized from methanol for further purification. The synthetic route is shown in Fig. 8 ▸.

Yellow solid, m.p. 487–488 K; yield: 65.32%. IR (*ν*, cm^−1^) 3211 (N—H, stretching), 3080 (C—H stretching, aromatic), 2968 (C—H stretching, aliphatic) 1697 (C=O stretching, ester), 1641 (C=O stretching, ketone). ^1^H NMR (DMSO-*d*_6_) *δ*: 0.84 (3H; *s*; 7-CH_3_), 1.00 (3H; *s*; 7-CH_3_), 1.31 [9H, *s*, C(CH_3_)_3_], 1.95–1.99 (2H; *d*; *J* = 16 Hz; quinoline H8), 2.13–2.16 (H; *d*; *J* = 16.1; quinoline H8), 2.25 (3H; *s*; 2-CH_3_), 2.26–2.30 (H; *d*; *J* = 16.95 quinoline H6), 2.37–2.41 (H; *d*; *J* =1 6.95 quinoline H6), 4.78 (1H; *s*; quinoline H4), 6.99–7.01 (2*H*, *d*, *J* = 8.5 Hz Ar—H3), 7.14 (1H; *t*; *J* = 74.4 Hz; OCHF_2_), 7.17–7.18 (2*H*, *d*, *J* = 10 Ar—H2), 8.99 (1*H*,*s*; NH). ^13^C NMR (DMSO-*d*_6_) *δ*: 18.7 (2-CH_3_), 27.0 (7-CH_3_), 28.3 [COOC(CH_3_)_3_], 29.4 (C-7), 32.0 (C-8), 36.2 (C-4), 50.6 (C-6), 79.2 [COOC(CH_3_)_3_], 105.4 (C-3), 110.0 (C-4a), 114.8 (C_3_’), 116.9, 118.4, 118.9 (OCHF_2_), 129.4 (C_2_’), 144.5 (C_1_’), 145.3 (C-2), 149.3 (C-8a), 150.0 (C_4_’), 166.7 [COOC(CH_3_)_3_], 194.6 (C-5). HRMS (ESI/Q-TOF) *m*/*z*: [*M* + H]^+^ Calculated for C_24_H_29_F_2_NO_4_ 433.2065; found 434.2328 (*M* + H).

## 6. Refinement

Crystal data, data collection and structure refinement details are summarized in Table 3 ▸. The N-bound H atom was located in a difference Fourier map and refined freely [N1—H1*N* = 0.91 (2) Å]. All C-bound H atoms were positioned geometrically [C—H = 0.95–1.00 Å] and refined using a riding model with *U*_iso_(H) = 1.2 or 1.5 *U*_eq_(C).

## Supplementary Material

Crystal structure: contains datablock(s) I. DOI: 10.1107/S2056989023005455/zn2030sup1.cif

Structure factors: contains datablock(s) I. DOI: 10.1107/S2056989023005455/zn2030Isup2.hkl

Supporting information file. DOI: 10.1107/S2056989023005455/zn2030Isup3.cml

CCDC reference: 2271384

Additional supporting information:  crystallographic information; 3D view; checkCIF report

## Figures and Tables

**Figure 4:**
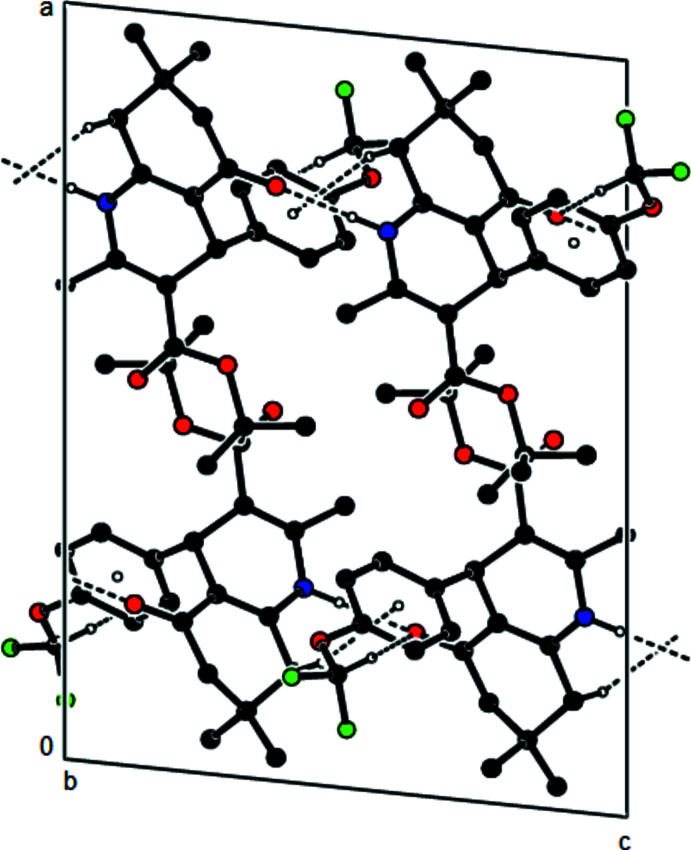
View of the mol­ecular packing along [010]. Hydrogen bonds are shown as dashed lines.

**Table 1:** Hydrogen-bond geometry (Å, °) *Cg*3 is the centroid of the C18–C23 ring.

*D*—H⋯*A*	*D*—H	H⋯*A*	*D*⋯*A*	*D*—H⋯*A*
N1—H1*N*⋯O1^i^	0.91 (2)	1.96 (2)	2.866 (2)	176.6 (18)
C12—H12*A*⋯O2	0.98	2.25	2.800 (2)	114
C16—H16*A*⋯O2	0.98	2.36	2.938 (2)	117
C17—H17*C*⋯O2	0.98	2.37	2.958 (3)	118
C20—H20*A*⋯F1	0.95	2.46	2.989 (2)	115
C24—H24*A*⋯O1^ii^	1.00	2.35	3.230 (2)	147
C2—H2*A*⋯*Cg*3^iii^	0.99	2.74	3.6959 (19)	162

Symmetry codes: (i) 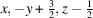 ; (ii) 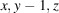 ; (iii) 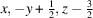 .

**Table 2:** Summary of short inter­atomic contacts (Å) in the title compound

H11*C*⋯H10*A*	2.49	2 − *x*, − 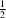 + *y*, 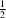 − *z*
F2⋯H19*A*	2.51	*x*, 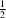 − *y*, 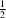 + *z*
O1⋯H24*A*	2.35	*x*, 1 + *y*, *z*
O1⋯H1*N*	1.96	*x*, 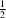 − *y*, − 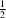 + *z*
H12*A*⋯O2	2.61	1 − *x*, 1 − *y*, −*z*
H15*A*⋯H12*A*	2.40	1 − *x*, 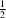 + *y*, 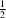 − *z*
H22*A*⋯H16*B*	2.38	1 − *x*, 1 − *y*, 1 − *z*

**Table 3:** Experimental details

Crystal data	
Chemical formula	C_24_H_29_F_2_NO_4_
*M*_r_	433.48
Crystal system, space group	Monoclinic, *P*2_1_/*c*
Temperature (K)	100
*a*, *b*, *c* (Å)	17.6062 (11), 9.7588 (7), 13.1509 (9)
β (°)	95.905 (2)
*V* (Å^3^)	2247.5 (3)
*Z*	4
Radiation type	Mo *K*α
μ (mm^−1^)	0.10
Crystal size (mm)	0.26 × 0.20 × 0.14

Data collection	
Diffractometer	Bruker D8 Quest with Photon 2 detector
Absorption correction	Multi-scan (*SADABS*; Bruker, 2018 ▸)
*T*_min_, *T*_max_	0.657, 0.746
No. of measured, independent and observed [*I* > 2σ(*I*)] reflections	31708, 4599, 3208
*R*_int_	0.111
(sin θ/λ)_max_ (Å^−1^)	0.625

Refinement	
*R*[*F* ^2^ > 2σ(*F* ^2^)], *wR*(*F* ^2^), *S*	0.046, 0.110, 1.02
No. of reflections	4599
No. of parameters	290
H-atom treatment	H atoms treated by a mixture of independent and constrained refinement
Δρ_max_, Δρ_min_ (e Å^−3^)	0.21, −0.26

Computer programs: *APEX2* and *SAINT* (Bruker, 2018 ▸), *SHELXT* (Sheldrick, 2015*a* ▸), *SHELXL* (Sheldrick, 2015*b* ▸), *ORTEP-3 for Windows* (Farrugia, 2012 ▸) and *PLATON* (Spek, 2020 ▸).

## supplementary crystallographic information

### Crystal data

**Table:**

C_24_H_29_F_2_NO_4_	*F*(000) = 920
*M**_r_* = 433.48	*D*_x_ = 1.281 Mg m^−^^3^
Monoclinic, *P*2_1_/*c*	Mo *K*α radiation, λ = 0.71073 Å
*a* = 17.6062 (11) Å	Cell parameters from 4059 reflections
*b* = 9.7588 (7) Å	θ = 2.3–30.3°
*c* = 13.1509 (9) Å	µ = 0.10 mm^−^^1^
β = 95.905 (2)°	*T* = 100 K
*V* = 2247.5 (3) Å^3^	Prism, colorless
*Z* = 4	0.26 × 0.20 × 0.14 mm

### Data collection

**Table:**

Bruker D8 Quest with Photon 2 detector diffractometer	3208 reflections with *I* > 2σ(*I*)
φ and ω scans	*R*_int_ = 0.111
Absorption correction: multi-scan (SADABS; Bruker, 2018)	θ_max_ = 26.4°, θ_min_ = 2.4°
*T*_min_ = 0.657, *T*_max_ = 0.746	*h* = −22→22
31708 measured reflections	*k* = −12→12
4599 independent reflections	*l* = −16→16

### Refinement

**Table:**

Refinement on *F*^2^	Primary atom site location: dual
Least-squares matrix: full	Secondary atom site location: difference Fourier map
*R*[*F*^2^ > 2σ(*F*^2^)] = 0.046	Hydrogen site location: mixed
*wR*(*F*^2^) = 0.110	H atoms treated by a mixture of independent and constrained refinement
*S* = 1.02	*w* = 1/[σ^2^(*F*_o_^2^) + (0.0445*P*)^2^ + 0.5785*P*] where *P* = (*F*_o_^2^ + 2*F*_c_^2^)/3
4599 reflections	(Δ/σ)_max_ = 0.001
290 parameters	Δρ_max_ = 0.21 e Å^−^^3^
0 restraints	Δρ_min_ = −0.26 e Å^−^^3^

### Special details

**Table:**

Geometry. All esds (except the esd in the dihedral angle between two l.s. planes) are estimated using the full covariance matrix. The cell esds are taken into account individually in the estimation of esds in distances, angles and torsion angles; correlations between esds in cell parameters are only used when they are defined by crystal symmetry. An approximate (isotropic) treatment of cell esds is used for estimating esds involving l.s. planes.

### Fractional atomic coordinates and isotropic or equivalent isotropic displacement parameters (Å^2^)

**Table:**

	*x*	*y*	*z*	*U*_iso_*/*U*_eq_	
F1	0.92250 (6)	0.20646 (12)	0.49817 (9)	0.0351 (3)	
F2	0.86051 (6)	0.08089 (11)	0.59614 (9)	0.0311 (3)	
O1	0.78594 (7)	0.91033 (13)	0.37607 (9)	0.0202 (3)	
O2	0.51155 (7)	0.57880 (16)	0.12973 (10)	0.0338 (4)	
O3	0.54282 (7)	0.65743 (13)	0.28906 (9)	0.0189 (3)	
O4	0.80976 (7)	0.27398 (13)	0.54548 (9)	0.0242 (3)	
N1	0.74130 (8)	0.64973 (15)	0.07479 (12)	0.0155 (3)	
H1N	0.7556 (11)	0.634 (2)	0.0116 (16)	0.027 (6)*	
C1	0.78375 (9)	0.73899 (17)	0.13677 (13)	0.0141 (4)	
C2	0.85366 (9)	0.79398 (18)	0.09469 (13)	0.0160 (4)	
H2A	0.838042	0.864711	0.042740	0.019*	
H2B	0.878600	0.718755	0.060078	0.019*	
C3	0.91163 (10)	0.85665 (18)	0.17650 (13)	0.0164 (4)	
C4	0.86773 (10)	0.94844 (18)	0.24516 (14)	0.0184 (4)	
H4A	0.903915	0.984065	0.301593	0.022*	
H4B	0.846852	1.027890	0.204631	0.022*	
C5	0.80277 (10)	0.87635 (17)	0.29047 (13)	0.0155 (4)	
C6	0.76121 (9)	0.77274 (17)	0.22958 (13)	0.0140 (4)	
C7	0.69507 (9)	0.69774 (18)	0.27054 (13)	0.0146 (4)	
H7A	0.666791	0.764154	0.310637	0.018*	
C8	0.64044 (10)	0.64486 (17)	0.18117 (13)	0.0148 (4)	
C9	0.66713 (9)	0.61265 (18)	0.09115 (13)	0.0153 (4)	
C10	0.96912 (11)	0.9421 (2)	0.12400 (15)	0.0245 (4)	
H10A	1.008339	0.977719	0.175509	0.037*	
H10B	0.942644	1.018719	0.087535	0.037*	
H10C	0.993191	0.884569	0.075323	0.037*	
C11	0.95397 (11)	0.7439 (2)	0.24056 (15)	0.0257 (5)	
H11A	0.990465	0.785721	0.292795	0.039*	
H11B	0.981403	0.685259	0.196126	0.039*	
H11C	0.917130	0.688629	0.273739	0.039*	
C12	0.62714 (10)	0.5386 (2)	0.00161 (14)	0.0209 (4)	
H12A	0.588198	0.477760	0.025035	0.031*	
H12B	0.664249	0.484180	−0.031788	0.031*	
H12C	0.602762	0.605142	−0.047102	0.031*	
C13	0.55921 (10)	0.62201 (18)	0.19487 (14)	0.0174 (4)	
C14	0.46412 (10)	0.64012 (19)	0.31875 (15)	0.0212 (4)	
C15	0.47276 (12)	0.6890 (2)	0.42836 (16)	0.0338 (5)	
H15A	0.487156	0.786090	0.430522	0.051*	
H15B	0.512507	0.635304	0.467906	0.051*	
H15C	0.424214	0.677487	0.457741	0.051*	
C16	0.44228 (11)	0.4893 (2)	0.31253 (16)	0.0272 (5)	
H16A	0.438276	0.459295	0.241063	0.041*	
H16B	0.393032	0.476330	0.339800	0.041*	
H16C	0.481471	0.435070	0.352732	0.041*	
C17	0.40767 (11)	0.7291 (2)	0.25328 (19)	0.0358 (6)	
H17A	0.424720	0.824723	0.257522	0.054*	
H17B	0.357177	0.721817	0.278004	0.054*	
H17C	0.404679	0.698213	0.182077	0.054*	
C18	0.72353 (10)	0.57950 (18)	0.34146 (13)	0.0151 (4)	
C19	0.77883 (10)	0.48904 (19)	0.31318 (14)	0.0207 (4)	
H19A	0.796943	0.499218	0.248000	0.025*	
C20	0.80838 (11)	0.38451 (19)	0.37706 (14)	0.0218 (4)	
H20A	0.846835	0.325229	0.356664	0.026*	
C21	0.78073 (10)	0.36846 (18)	0.47084 (14)	0.0185 (4)	
C22	0.72373 (10)	0.45291 (19)	0.49978 (14)	0.0192 (4)	
H22A	0.703948	0.439405	0.563564	0.023*	
C23	0.69559 (10)	0.55749 (19)	0.43512 (13)	0.0179 (4)	
H23A	0.656394	0.615290	0.455307	0.021*	
C24	0.85104 (11)	0.16566 (19)	0.51508 (15)	0.0223 (4)	
H24A	0.824329	0.118997	0.453797	0.027*	

### Atomic displacement parameters (Å^2^)

**Table:**

	*U*^11^	*U*^22^	*U*^33^	*U*^12^	*U*^13^	*U*^23^
F1	0.0253 (6)	0.0380 (7)	0.0430 (8)	−0.0014 (5)	0.0079 (5)	0.0154 (6)
F2	0.0389 (7)	0.0244 (6)	0.0305 (7)	0.0009 (5)	0.0062 (5)	0.0127 (5)
O1	0.0250 (7)	0.0224 (7)	0.0140 (7)	−0.0035 (6)	0.0055 (5)	−0.0038 (5)
O2	0.0210 (7)	0.0590 (10)	0.0214 (8)	−0.0140 (7)	0.0026 (6)	−0.0078 (7)
O3	0.0145 (6)	0.0228 (7)	0.0204 (7)	−0.0024 (5)	0.0062 (5)	−0.0030 (6)
O4	0.0342 (8)	0.0217 (7)	0.0169 (7)	0.0053 (6)	0.0039 (6)	0.0042 (6)
N1	0.0168 (8)	0.0190 (8)	0.0113 (8)	−0.0013 (6)	0.0045 (6)	−0.0025 (6)
C1	0.0139 (9)	0.0133 (9)	0.0149 (9)	0.0020 (7)	0.0001 (7)	0.0013 (7)
C2	0.0175 (9)	0.0158 (9)	0.0156 (9)	0.0005 (7)	0.0051 (7)	0.0010 (7)
C3	0.0165 (9)	0.0166 (9)	0.0166 (9)	−0.0011 (7)	0.0035 (7)	−0.0002 (8)
C4	0.0200 (10)	0.0175 (9)	0.0179 (10)	−0.0043 (8)	0.0036 (8)	−0.0018 (8)
C5	0.0173 (9)	0.0146 (9)	0.0147 (9)	0.0031 (7)	0.0014 (7)	0.0022 (7)
C6	0.0129 (9)	0.0156 (9)	0.0136 (9)	0.0011 (7)	0.0017 (7)	0.0017 (7)
C7	0.0150 (9)	0.0158 (9)	0.0135 (9)	−0.0009 (7)	0.0040 (7)	−0.0001 (7)
C8	0.0164 (9)	0.0138 (9)	0.0141 (9)	−0.0002 (7)	0.0008 (7)	0.0015 (7)
C9	0.0150 (9)	0.0150 (9)	0.0158 (9)	−0.0006 (7)	0.0010 (7)	0.0025 (7)
C10	0.0211 (10)	0.0255 (11)	0.0287 (11)	−0.0057 (8)	0.0107 (8)	−0.0022 (9)
C11	0.0203 (10)	0.0299 (11)	0.0265 (11)	0.0033 (9)	−0.0001 (8)	0.0027 (9)
C12	0.0220 (10)	0.0240 (10)	0.0167 (10)	−0.0049 (8)	0.0018 (8)	−0.0031 (8)
C13	0.0185 (10)	0.0183 (10)	0.0156 (9)	−0.0012 (7)	0.0024 (8)	0.0009 (8)
C14	0.0141 (9)	0.0236 (10)	0.0280 (11)	−0.0036 (8)	0.0119 (8)	−0.0044 (8)
C15	0.0263 (11)	0.0417 (13)	0.0362 (13)	−0.0094 (10)	0.0172 (10)	−0.0157 (11)
C16	0.0265 (11)	0.0233 (11)	0.0347 (12)	−0.0060 (9)	0.0169 (9)	−0.0047 (9)
C17	0.0184 (10)	0.0345 (12)	0.0560 (16)	0.0035 (9)	0.0117 (10)	0.0062 (11)
C18	0.0152 (9)	0.0173 (9)	0.0128 (9)	−0.0051 (7)	0.0016 (7)	−0.0010 (7)
C19	0.0262 (10)	0.0224 (10)	0.0146 (10)	0.0007 (8)	0.0072 (8)	0.0007 (8)
C20	0.0259 (10)	0.0212 (10)	0.0191 (10)	0.0033 (8)	0.0062 (8)	−0.0008 (8)
C21	0.0223 (10)	0.0163 (10)	0.0165 (9)	−0.0032 (8)	−0.0003 (8)	0.0010 (8)
C22	0.0204 (10)	0.0252 (10)	0.0126 (9)	−0.0049 (8)	0.0051 (7)	0.0015 (8)
C23	0.0149 (9)	0.0230 (10)	0.0161 (9)	−0.0027 (7)	0.0035 (7)	−0.0019 (8)
C24	0.0257 (11)	0.0186 (10)	0.0228 (10)	−0.0026 (8)	0.0032 (8)	0.0046 (8)

### Geometric parameters (Å, º)

**Table:**

F1—C24	1.360 (2)	C10—H10B	0.9800
F2—C24	1.346 (2)	C10—H10C	0.9800
O1—C5	1.238 (2)	C11—H11A	0.9800
O2—C13	1.212 (2)	C11—H11B	0.9800
O3—C13	1.346 (2)	C11—H11C	0.9800
O3—C14	1.487 (2)	C12—H12A	0.9800
O4—C24	1.366 (2)	C12—H12B	0.9800
O4—C21	1.404 (2)	C12—H12C	0.9800
N1—C1	1.363 (2)	C14—C15	1.511 (3)
N1—C9	1.393 (2)	C14—C17	1.519 (3)
N1—H1N	0.91 (2)	C14—C16	1.521 (3)
C1—C6	1.362 (2)	C15—H15A	0.9800
C1—C2	1.500 (2)	C15—H15B	0.9800
C2—C3	1.532 (2)	C15—H15C	0.9800
C2—H2A	0.9900	C16—H16A	0.9800
C2—H2B	0.9900	C16—H16B	0.9800
C3—C10	1.530 (2)	C16—H16C	0.9800
C3—C11	1.532 (2)	C17—H17A	0.9800
C3—C4	1.536 (2)	C17—H17B	0.9800
C4—C5	1.516 (2)	C17—H17C	0.9800
C4—H4A	0.9900	C18—C23	1.389 (2)
C4—H4B	0.9900	C18—C19	1.393 (2)
C5—C6	1.442 (2)	C19—C20	1.388 (3)
C6—C7	1.520 (2)	C19—H19A	0.9500
C7—C8	1.530 (2)	C20—C21	1.381 (3)
C7—C18	1.535 (2)	C20—H20A	0.9500
C7—H7A	1.0000	C21—C22	1.382 (3)
C8—C9	1.355 (2)	C22—C23	1.387 (3)
C8—C13	1.477 (2)	C22—H22A	0.9500
C9—C12	1.495 (2)	C23—H23A	0.9500
C10—H10A	0.9800	C24—H24A	1.0000
			
C13—O3—C14	120.42 (13)	C9—C12—H12B	109.5
C24—O4—C21	118.04 (14)	H12A—C12—H12B	109.5
C1—N1—C9	122.61 (15)	C9—C12—H12C	109.5
C1—N1—H1N	117.9 (13)	H12A—C12—H12C	109.5
C9—N1—H1N	116.9 (12)	H12B—C12—H12C	109.5
C6—C1—N1	119.88 (16)	O2—C13—O3	122.80 (16)
C6—C1—C2	124.75 (16)	O2—C13—C8	125.12 (17)
N1—C1—C2	115.38 (15)	O3—C13—C8	112.06 (15)
C1—C2—C3	113.36 (14)	O3—C14—C15	102.09 (14)
C1—C2—H2A	108.9	O3—C14—C17	111.05 (15)
C3—C2—H2A	108.9	C15—C14—C17	110.84 (17)
C1—C2—H2B	108.9	O3—C14—C16	109.44 (14)
C3—C2—H2B	108.9	C15—C14—C16	110.92 (17)
H2A—C2—H2B	107.7	C17—C14—C16	112.07 (16)
C10—C3—C11	109.42 (15)	C14—C15—H15A	109.5
C10—C3—C2	108.94 (14)	C14—C15—H15B	109.5
C11—C3—C2	110.54 (15)	H15A—C15—H15B	109.5
C10—C3—C4	110.11 (15)	C14—C15—H15C	109.5
C11—C3—C4	109.96 (15)	H15A—C15—H15C	109.5
C2—C3—C4	107.85 (14)	H15B—C15—H15C	109.5
C5—C4—C3	113.96 (14)	C14—C16—H16A	109.5
C5—C4—H4A	108.8	C14—C16—H16B	109.5
C3—C4—H4A	108.8	H16A—C16—H16B	109.5
C5—C4—H4B	108.8	C14—C16—H16C	109.5
C3—C4—H4B	108.8	H16A—C16—H16C	109.5
H4A—C4—H4B	107.7	H16B—C16—H16C	109.5
O1—C5—C6	122.52 (16)	C14—C17—H17A	109.5
O1—C5—C4	119.59 (15)	C14—C17—H17B	109.5
C6—C5—C4	117.87 (15)	H17A—C17—H17B	109.5
C1—C6—C5	119.29 (15)	C14—C17—H17C	109.5
C1—C6—C7	120.37 (15)	H17A—C17—H17C	109.5
C5—C6—C7	120.29 (15)	H17B—C17—H17C	109.5
C6—C7—C8	109.52 (14)	C23—C18—C19	117.37 (16)
C6—C7—C18	111.30 (13)	C23—C18—C7	122.12 (16)
C8—C7—C18	110.69 (14)	C19—C18—C7	120.51 (16)
C6—C7—H7A	108.4	C20—C19—C18	122.20 (17)
C8—C7—H7A	108.4	C20—C19—H19A	118.9
C18—C7—H7A	108.4	C18—C19—H19A	118.9
C9—C8—C13	119.93 (16)	C21—C20—C19	118.65 (17)
C9—C8—C7	120.16 (15)	C21—C20—H20A	120.7
C13—C8—C7	119.83 (15)	C19—C20—H20A	120.7
C8—C9—N1	119.31 (16)	C20—C21—C22	120.74 (17)
C8—C9—C12	128.53 (16)	C20—C21—O4	124.23 (16)
N1—C9—C12	112.15 (15)	C22—C21—O4	114.93 (16)
C3—C10—H10A	109.5	C21—C22—C23	119.59 (17)
C3—C10—H10B	109.5	C21—C22—H22A	120.2
H10A—C10—H10B	109.5	C23—C22—H22A	120.2
C3—C10—H10C	109.5	C22—C23—C18	121.37 (17)
H10A—C10—H10C	109.5	C22—C23—H23A	119.3
H10B—C10—H10C	109.5	C18—C23—H23A	119.3
C3—C11—H11A	109.5	F2—C24—F1	105.56 (14)
C3—C11—H11B	109.5	F2—C24—O4	105.67 (15)
H11A—C11—H11B	109.5	F1—C24—O4	110.52 (15)
C3—C11—H11C	109.5	F2—C24—H24A	111.6
H11A—C11—H11C	109.5	F1—C24—H24A	111.6
H11B—C11—H11C	109.5	O4—C24—H24A	111.6
C9—C12—H12A	109.5		
			
C9—N1—C1—C6	14.1 (2)	C7—C8—C9—C12	168.75 (17)
C9—N1—C1—C2	−165.54 (15)	C1—N1—C9—C8	−12.6 (3)
C6—C1—C2—C3	18.1 (2)	C1—N1—C9—C12	167.69 (16)
N1—C1—C2—C3	−162.30 (14)	C14—O3—C13—O2	2.1 (3)
C1—C2—C3—C10	−165.09 (15)	C14—O3—C13—C8	−179.04 (14)
C1—C2—C3—C11	74.64 (19)	C9—C8—C13—O2	−2.2 (3)
C1—C2—C3—C4	−45.59 (19)	C7—C8—C13—O2	−178.97 (18)
C10—C3—C4—C5	172.96 (15)	C9—C8—C13—O3	179.00 (16)
C11—C3—C4—C5	−66.40 (19)	C7—C8—C13—O3	2.2 (2)
C2—C3—C4—C5	54.20 (19)	C13—O3—C14—C15	178.94 (16)
C3—C4—C5—O1	147.75 (16)	C13—O3—C14—C17	−62.9 (2)
C3—C4—C5—C6	−34.2 (2)	C13—O3—C14—C16	61.4 (2)
N1—C1—C6—C5	−174.53 (15)	C6—C7—C18—C23	134.13 (17)
C2—C1—C6—C5	5.1 (3)	C8—C7—C18—C23	−103.82 (18)
N1—C1—C6—C7	8.1 (2)	C6—C7—C18—C19	−46.2 (2)
C2—C1—C6—C7	−172.29 (15)	C8—C7—C18—C19	75.88 (19)
O1—C5—C6—C1	−178.91 (16)	C23—C18—C19—C20	−3.1 (3)
C4—C5—C6—C1	3.1 (2)	C7—C18—C19—C20	177.17 (16)
O1—C5—C6—C7	−1.5 (3)	C18—C19—C20—C21	1.3 (3)
C4—C5—C6—C7	−179.50 (15)	C19—C20—C21—C22	1.3 (3)
C1—C6—C7—C8	−27.6 (2)	C19—C20—C21—O4	−174.90 (16)
C5—C6—C7—C8	155.05 (15)	C24—O4—C21—C20	−20.1 (3)
C1—C6—C7—C18	95.14 (19)	C24—O4—C21—C22	163.51 (16)
C5—C6—C7—C18	−82.24 (19)	C20—C21—C22—C23	−1.9 (3)
C6—C7—C8—C9	29.0 (2)	O4—C21—C22—C23	174.61 (15)
C18—C7—C8—C9	−94.12 (19)	C21—C22—C23—C18	0.0 (3)
C6—C7—C8—C13	−154.26 (15)	C19—C18—C23—C22	2.5 (3)
C18—C7—C8—C13	82.65 (19)	C7—C18—C23—C22	−177.84 (15)
C13—C8—C9—N1	172.33 (15)	C21—O4—C24—F2	−169.91 (14)
C7—C8—C9—N1	−10.9 (2)	C21—O4—C24—F1	76.36 (19)
C13—C8—C9—C12	−8.0 (3)		

### Hydrogen-bond geometry (Å, º)

*Cg*3 is the centroid of the C18–C23 ring.

**Table:**

*D*—H···*A*	*D*—H	H···*A*	*D*···*A*	*D*—H···*A*
N1—H1*N*···O1^i^	0.91 (2)	1.96 (2)	2.866 (2)	176.6 (18)
C12—H12*A*···O2	0.98	2.25	2.800 (2)	114
C16—H16*A*···O2	0.98	2.36	2.938 (2)	117
C17—H17*C*···O2	0.98	2.37	2.958 (3)	118
C20—H20*A*···F1	0.95	2.46	2.989 (2)	115
C24—H24*A*···O1^ii^	1.00	2.35	3.230 (2)	147
C2—H2*A*···*Cg*3^iii^	0.99	2.74	3.6959 (19)	162

Symmetry codes: (i) *x*, −*y*+3/2, *z*−1/2; (ii) *x*, *y*−1, *z*; (iii) *x*, −*y*+1/2, *z*−3/2.

## References

[bb1] Bruker (2018). *APEX2*, *SAINT* and *SADABS*. Bruker AXS Inc., Madison, Wisconsin, USA.

[bb2] Çetin, G., Çetin, B., Çolak, B., Aşan, M., Birlik Demirel, G., Cansaran-Duman, D., Akçelik, N. & Şimşek, R. (2022). *J. Res. Pharm.* **26**, 219–230.

[bb3] Cremer, D. & Pople, J. A. (1975). *J. Am. Chem. Soc.* **97**, 1354–1358.

[bb4] Elwahy, A. H. M., Eid, E. M., Abdel-Latif, S. A., Hassaneen, H. M. E. & Abdelhamid, I. A. (2022). *Polycyclic Aromat. Compd.* pp. 1–30.

[bb5] Farrugia, L. J. (2012). *J. Appl. Cryst.* **45**, 849–854.

[bb6] Furman, D., Campisi, J., Verdin, E., Carrera-Bastos, P., Targ, S., Franceschi, C., Ferrucci, L., Gilroy, D. W., Fasano, A., Miller, G. W., Miller, A. H., Mantovani, A., Weyand, C. M., Barzilai, N., Goronzy, J. J., Rando, T. A., Effros, R. B., Lucia, A., Kleinstreuer, N. & Slavich, G. M. (2019). *Nat. Med.* **25**, 1822–1832. 10.1038/s41591-019-0675-0 PMC7147972 31806905

[bb7] Ghosh, S., Saikh, F., Das, J. & Pramanik, A. K. (2013). *Tetrahedron Lett.* **54**, 58–62.

[bb8] Groom, C. R., Bruno, I. J., Lightfoot, M. P. & Ward, S. C. (2016). *Acta Cryst.* B**72**, 171–179. 10.1107/S2052520616003954 PMC4822653 27048719

[bb9] Gündüz, M. G., Butcher, R. J., Öztürk Yildirim, S., El-Khouly, A., Şafak, C. & Şimşek, R. (2012). *Acta Cryst.* E**68**, o3404–o3405. 10.1107/S1600536812046909 PMC3588994 23476230

[bb10] Khot, S., Auti, P. B. & Khedkar, S. A. (2021). *Mini Rev. Med. Chem.* **21**, 135–149. 10.2174/1389557520666200807130215 32767934

[bb12] Linden, A., Gündüz, M. G., Şimşek, R. & Şafak, C. (2006). *Acta Cryst.* C**62**, o227–o230. 10.1107/S0108270106007530 16598150

[bb13] Linden, A., Şafak, C., Şimşek, R. & Gündüz, M. G. (2011). *Acta Cryst.* C**67**, o80–o84. 10.1107/S0108270111003362 21285508

[bb14] Linden, A., Şimşek, R., Gündüz, M. & Şafak, C. (2005). *Acta Cryst.* C**61**, o731–o734. 10.1107/S0108270105037066 16330861

[bb15] Mookiah, P., Rajesh, K., Narasimhamurthy, T., Vijayakumar, V. & Srinivasan, N. (2009). *Acta Cryst.* E**65**, o2664. 10.1107/S1600536809039877 PMC2971334 21578275

[bb16] Öztürk Yildirim, S., Butcher, R. J., Gündüz, M. G., El-Khouly, A., Şimşek, R. & Şafak, C. (2013). *Acta Cryst.* E**69**, o40–o41. 10.1107/S1600536812047976 PMC3588325 23476426

[bb17] Sheldrick, G. M. (2015*a*). *Acta Cryst.* A**71**, 3–8.

[bb18] Sheldrick, G. M. (2015*b*). *Acta Cryst.* C**71**, 3–8.

[bb19] Spackman, P. R., Turner, M. J., McKinnon, J. J., Wolff, S. K., Grimwood, D. J., Jayatilaka, D. & Spackman, M. A. (2021). *J. Appl. Cryst.* **54**, 1006–1011. 10.1107/S1600576721002910 PMC8202033 34188619

[bb20] Spek, A. L. (2020). *Acta Cryst.* E**76**, 1–11. 10.1107/S2056989019016244 PMC6944088 31921444

[bb21] Steiger, S. A., Monacelli, A. J., Li, C., Hunting, J. L. & Natale, N. R. (2014). *Acta Cryst.* C**70**, 790–795. 10.1107/S2053229614015617 PMC4174017 25093361

[bb11] Tu, Z., Zhong, Y., Hu, H., Shao, D., Haag, R., Schirner, M., Lee, J., Sullenger, B. & Leong, K. W. (2022). *Nat. Rev. Mater.* 557–574. 10.1038/s41578-022-00426-z PMC8884103 35251702

[bb22] Yıldırım, S. Ö., Akkurt, M., Çetin, G., Şimşek, R., Butcher, R. J. & Bhattarai, A. (2022). *Acta Cryst.* E**78**, 798–803. 10.1107/S2056989022007022 PMC9361379 35974826

[bb23] Yıldırım, S. Ö., Akkurt, M., Çetin, G., Şimşek, R., Butcher, R. J. & Bhattarai, A. (2023). *Acta Cryst.* E**79**, 187–191. 10.1107/S205698902300141X PMC9993909 36909987

